# Genome-Wide Association Analysis of Gibberellin Sensitivity for Panicle Exsertion Length in Rice and Candidate Gene Identification

**DOI:** 10.3390/plants15132063

**Published:** 2026-07-02

**Authors:** Yulong Li, Li Zhao, Xiyong Zhao, Ze Liu, Quankai Jing, Jun Li, Fanfan Ren, Delin Hong, Songbai Rong

**Affiliations:** 1 Anhui Provincial Key Laboratory of Crop Quality Improvement, Institute of Crop Research, Anhui Academy of Agricultural Sciences, Hefei 230001, China; 2 State Key Laboratory of Crop Genetics and Germplasm Enhancement, Nanjing Agricultural University, Nanjing 210095, China

**Keywords:** panicle exsertion length, gibberellin sensitivity, genome-wide association study, RLCK kinase, hybrid rice seed production

## Abstract

Insufficient panicle exsertion length (PEL) in hybrid rice production requires heavy gibberellin (GA_3_) application, increasing costs and pollution. This study explored genetic resources regulating GA_3_ sensitivity to enable low-GA_3_-dependent breeding. A panel of 173 rice accessions was treated with GA_3_ (0.1 mg/plant) over two years. The GA_3_ sensitivity index (PEL_GSI_) was calculated as (PEL_GA3_ − PEL_n_)/PEL_n_. A genome-wide association study identified seven QTLs controlling PEL_GSI_, with *qPEL_GSI_3.2* on chromosome 3 being the major locus. Through linkage disequilibrium analysis, non-synonymous mutation screening, and qRT-PCR, *LOC_Os03g15770* (encoding a receptor-like cytoplasmic kinase) was identified as a promising candidate gene. Haplotype analysis showed that Hap 1 is the elite allele, conferring significantly higher PEL_GSI_ (2.69%) than Hap 2 (1.41%). This study reports the genetic locus and candidate gene regulating exogenous GA_3_ sensitivity in rice, providing new resources for marker-assisted breeding of low-GA_3_-dependent hybrid rice parents.

## 1. Introduction

Rice panicle exsertion length (PEL) is one of the important factors affecting the yield of hybrid rice seed production. In China, hybrid rice accounts for approximately 50% of the total rice planting area, with over 18 million hectares annually, and contributes significantly to national food security [1]. Major commercial hybrids such as Liangyoupeijiu, Y-liangyou 1, and Fengliangyou 4 are widely cultivated in the Yangtze River basin. However, many of these hybrids, particularly those based on sterile lines such as Zhenshan 97A and Peiai 64S, suffer from insufficient PEL, necessitating large-scale GA_3_ application during seed production [2]. This context highlights the practical urgency of identifying the genetic factors that enhance GA_3_ sensitivity and reduce exogenous hormone dependency.

The failure of the panicle to fully exsert itself in the sterile line is the main factor limiting the propagation of the sterile line and the increase of hybrid rice seed production [3]. When these sterile lines are widely promoted and applied in production, due to their short PEL, large amounts of gibberellin need to be sprayed, which not only increases seed production costs but also exacerbates environmental pollution caused by chemicals [2]. Therefore, breeding rice sterile lines with moderate PEL through genetic improvement has attracted widespread attention from rice breeders.

Rutger and Carnahan [4] reported the discovery of a recessive tall mutant 79:4512 with elongated uppermost internodes in the F_3_ population of the japonica rice Mg/Terso and named the recessive tall gene *eui*. It is believed that the *eui* gene can serve as the fourth genetic factor in hybrid rice seed production (relative to the sterile line, maintainer line, and restorer line). Shen et al. [5] first introduced the recessive gene *eui* controlling PEL into Zhenshan 97A and bred the non-collared wild abortive type sterile lines. Zhenchang A and B. Wang et al. [6] introduced the long PEL gene *eui* from”02428h” into Peiai 64S, resulting in the conversion of P8hS carrying the *eui* gene. However, these materials have not been widely used in actual production. Therefore, it is necessary to exploit QTLs and genes controlling PEL using different genetic materials and to breed long PEL varieties through marker-assisted selection.

PEL is a complex quantitative trait [7]. Currently, approximately 14 mutants have been reported, including *A864*, *shp1*, *shp2*, *shp3*, *shp4*, *shp5*, *shp6*, *M893*, SUI-family (*sui1*, *sui2*, *sui3*, and *sui4*), *esp1*, and *esp2* [8,9,10,11,12,13]. Zhu [9] detected *SHP5* and *SHP6* on chromosomes 4 and 2, respectively. Duan et al. [14] detected *esp1* on chromosome 11. Guan et al. [10] reported that *esp2* was finely mapped into 14 kb on chromosome 1, and *LOC_Os01g02890* was the candidate gene.

To our knowledge, more than 170 QTLs controlling PEL have been mapped from different populations [7,15,16,17,18,19,20,21,22,23,24]. Despite so many QTLs having been detected, only 12 genes have been cloned, namely, *EUI1* [25,26], *EUI2* [27], SUI-family (*SUI1*, *SUI2*, *SUI3*, and *SUI4*) [12,13], *HOX12* [18], *pds1* [28], *ACE1* [29], *DEC1* [29], *PEL9* [21], and *EPE1* [24]. *EUI1* encodes a cytochrome P450 monooxygenase CYP714D1 and negatively regulates GA-mediated cell elongation in the uppermost internode of rice [25,26]. *EUI2* encodes an epoxide hydrolase and is involved in the dehydrogenation of reactive GA [27]. SUI family genes encode phospholipidserine synthase, which can regulate the development of IM, controlling internode elongation and the cell expansion of the spikelet axis [12,13]. The rice *pds1* locus genetically interacts with its partner to cause panicle exsertion defects and ectopic tillers in spikelets [28]. Rice *HOX12* regulates panicle exsertion by directly modulating the expression of EUI1 [18]. *ACE1* encodes a protein of unknown function that enables cell division in the internode meristem region to elongate the internode. *DEC1* encodes a zinc finger transcription factor, and upregulation of *DEC1* expression inhibits internode elongation while downregulation of *DEC1* promotes internode elongation [29]. *PEL9* encodes the flavonol synthase protein, and the elite allele *PEL9*^GG^ can increase hybrid F_1_ seed production by 14.81% [21]. *EPE1* encodes gibberellin 2-oxidase, which can regulate the transcription of α-amylase encoding genes through GA signaling [24]. Despite these advances, GA_3_-sensitive rice germplasm resources and the genes controlling GA_3_ sensitivity have not yet been reported.

In this study, we applied exogenous GA_3_ to 173 rice germplasm accessions at stages 4–5 of young panicle differentiation; the sensitivity index of 173 accessions was used as the trait value, and genome-wide association analysis was performed using a mixed linear model combined with single nucleotide polymorphism (SNP) data to detect SNP markers significantly associated with trait variation and identify favorable candidate genes sensitive to exogenous gibberellin. These results will provide genetic and vector information for improving the outcrossing trait, increasing the yield of hybrid rice seed production, and achieving simplified hybrid rice seed production.

## 2. Results

### 2.1. Phenotypic Analysis

**Figure 1:**
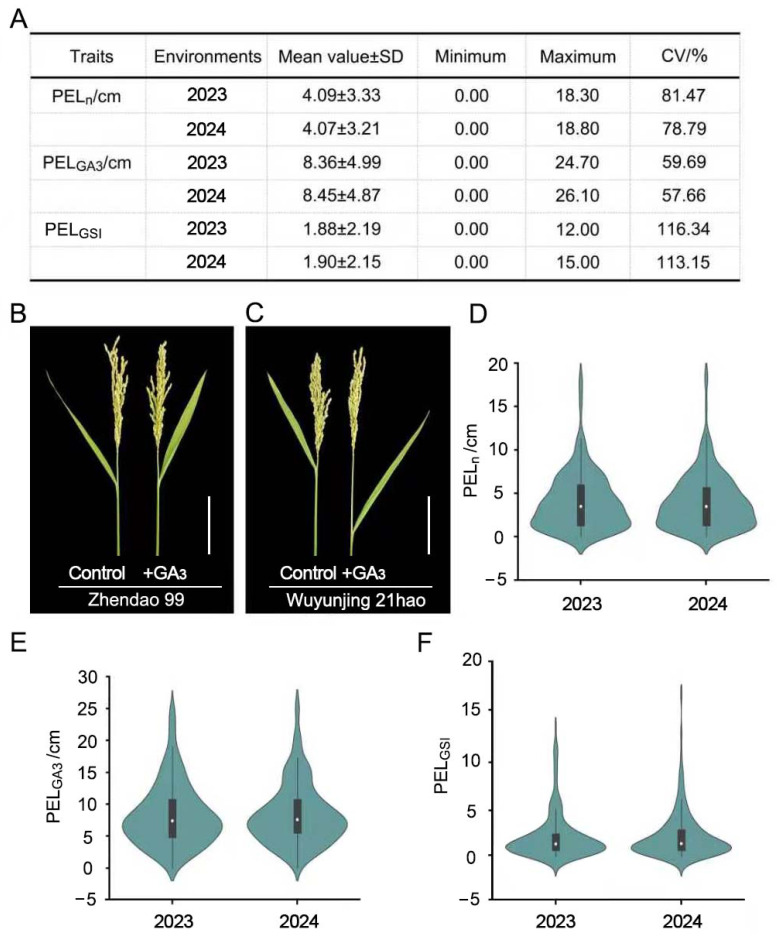
Phenotypic diversity of PEL in rice. (**A**) Phenotypic statistics of PEL in 2023 and 2024. (**B**) Panicle morphology of Zhendao 99 with low PEL in normal and treatment conditions. Scale bar, 5 cm. (**C**) Panicle morphology of Wuyunjing 21hao with high PEL in normal and treatment conditions. Scale bar, 5 cm. (**D**) Variation of PEL_n_ among the 173 rice accessions in 2023 and 2024. (**E**) Variation of PEL_GA3_ among the 173 rice accessions in 2023 and 2024. (**F**) Variation in PEL_GSI_ among the 173 rice accessions in 2023 and 2024. PEL: panicle exsertion length; PEL_n_: panicle exsertion length under normal conditions; PEL_GA3_: panicle exsertion length under GA_3_ treatment condition; PEL_GSI_: GA_3_ sensitivity index of PEL.

Figure 1A lists the basic statistical information such as maximum, minimum, mean value, and coefficient of variation (CV). For the material Zhendao 99, the mean value of PEL_n_ was 5.70 cm and the mean value of PEL_GA3_ was 8.65 cm, giving a smaller PEL_D_ (2.95 cm) (Figure 1B). For the material Wuyunjing 21hao, the mean value of PEL_n_ was 6.20 cm and the mean value of PEL_GA3_ was 24.00 cm, giving the maximum PEL_D_ (17.8 cm) (Figure 1C). For the PEL_n_ trait, the maximum value of PEL_n_ was 18.55 cm, and the minimum value was 0.00 cm among the two environments (Figure 1A,D). The CV of PEL_n_ ranged from 78.79% to 81.47% across the two environments. For the PEL_GA3_ trait, the maximum value of PEL_GA3_ was 25.40 cm and the minimum value was 0.00 cm among two environments, with CV ranging from 57.66% to 59.69% (Figure 1A,E). Compared with PEL_n_, the number of materials with a long PEL was increased (Figure 1E). For the PEL_GSI_ trait, the mean value was 1.88 ± 2.19 in 2023 and 1.90 ± 2.15 in 2024, with CV ranging from 113.15% to 116.34% (Figure 1A,F). More than half of the accessions had low PEL_GSI_ values (Figure 1F).

To evaluate the stability of the PEL_GSI_ trait across environments, we performed a two-way analysis of variance with genotype, year, and their interaction, as factors (Supplementary Table S1). The results revealed that genotype effects were highly significant for PEL_n_, PEL_GA3_, PEL_GSI_, and PEL_D_, confirming extensive genetic diversity for GA_3_ sensitivity (Supplementary Table S1). Significant genotype × environment interactions were detected for all traits (*p* < 0.01), while the environment main effect was not significant (*p* > 0.05), indicating that the traits were primarily under genetic control and stable across the two years (Supplementary Table S1).

### 2.2. Identification of QTLs by GWAS

**Figure 2:**
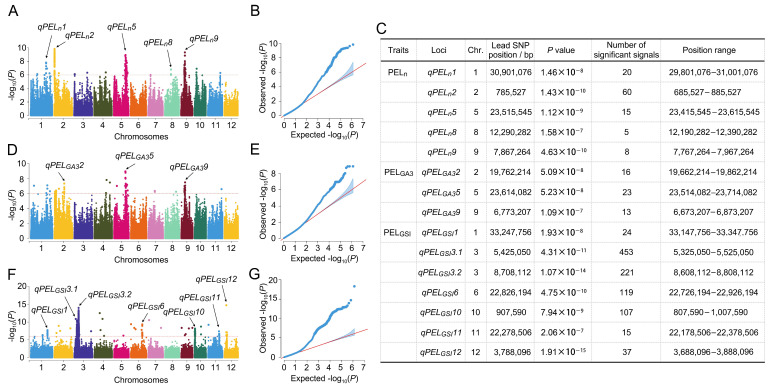
Identification of QTLs by GWAS in rice. (**A**) Manhattan plots for PEL_n_ in the whole population of rice accessions. (**B**) QQ-plot for PEL_n_. (**C**) Information on the identified QTLs for PEL_n_, PEL_GA3_, and PEL_GSI_. (**D**) Manhattan plots for PEL_GA3_ in the whole population of rice accessions. (**E**) QQ-plot for PEL_GA3_. (**F**) Manhattan plots for PEL_GSI_ in the whole population of rice accessions. (**G**) QQ-plot for PEL_GSI_. The *Y*-axis indicates negative log_10_ transformed *p* values, and dots above the red line show the significant SNPs in the QTL region. The black arrows indicate the QTL identified. PEL_n_: panicle exsertion length under normal condition; PEL_GA3_: panicle exsertion length under GA_3_ treatment conditions; PEL_GSI_: GA_3_ sensitivity index of PEL.

GWAS was conducted to analyze the natural variation of PEL in response to GA_3_ sensitivity, and Manhattan plots and QQ plots were generated to illustrate the significant SNPs (Figure 2). The QQ plot exhibited an early upward deviation of observed *p*-values from the expected null distribution, revealing mild genomic inflation in this GWAS panel. This inflation signal is widely observed in crop natural populations, which mainly arises from inherent population stratification and subtle pairwise kinship among the 173 rice accessions. To mitigate false positive associations triggered by population structure, we incorporated PCA-derived population covariates and a kinship matrix (K matrix) into the mixed linear model during association mapping, which effectively controlled the confounding genetic background effects.

For the PEL_n_ trait, a total of five QTLs were detected, which were located on chromosomes 1, 2, 5, 8, and 9 (Figure 2A–C). In the region of *qPEL_n_2*, there existed 60 significant SNPs. The QTLs *qPEL_n_1*, *qPEL_n_2*, *qPEL_n_5* and *qPEL_n_9* were simultaneously detected in 2023 and 2024 (Figure S1).

For the PEL_GA3_ trait, three QTLs were identified, locating on chromosomes 2, 5, and 9 (Figure 2C–E). In the region of *qPEL_GA3_5*, there existed 23 significant SNPs. The QTLs *qPEL_GA3_2.2*, *qPEL_GA3_5*, and *qPEL_GA3_9* were simultaneously detected in 2023 and 2024 (Figure S2). For the PEL_GSI_ trait, seven QTLs were identified, locating on chromosomes 1, 3, 6, 10, 11, and 12 (Figure 2C,F,G). In the region of *qPEL_GSI_3.2*, there existed 221 significant SNPs. The QTL *qPEL_GSI_3.2* was simultaneously detected in 2023 and 2024 (Figure S3).

To rule out the possibility that the ratio-based index (PEL_GSI_) might be biased by extremely small PEL_n_ values, we also performed GWAS using PEL_D_ as the trait (Figure S4). The major QTL *qPEL_GSI_3.2* was still detected at the same genomic region (leading SNP), demonstrating that our results are robust and not dependent on the denominator. And the *qPEL_GSI_3.2* was considered a major QTL for *PEL_GSI_*. Therefore, *qPEL_GSI_3.2* was selected for further study.

### 2.3. LOC_Os03g15770 Was Identified as a Candidate Gene

**Figure 3:**
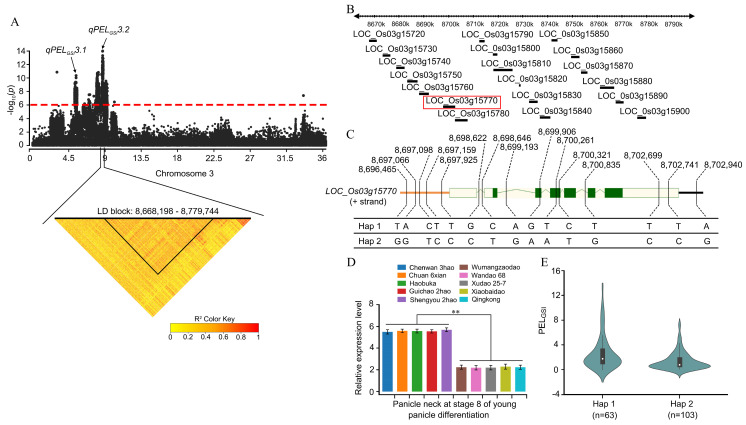
Identification of the candidate genes for the QTL *qPEL_GSI_3.2*. (**A**) Identification of haplotype block of *qPEL_GSI_3.2*. The *Y*-axis indicates negative log_10_ transformed *p* values and dots above the red line show the significant SNPs in the QTL region. Pairwise LD was determined by calculation of *r*^2^ (the square of the correlation coefficient between SNP states). (**B**) Identification of candidate genes in the region of *qPEL_GSI_3.2.* The candidate gene was selected in the red box. (**C**) Haplotypes of *LOC_Os03g15770* associated with PEL_GSI_ in rice. (**D**) Expression analysis of candidate gene *LOC_Os03g15770* in different materials. The relative expression values were normalized to the rice UBQ gene. (**E**) Box-plots of PEL_GSI_ in accessions containing the different haplotypes. Error bars indicate standard deviation, and asterisks indicate significant differences using the Student’s *t* test (** *p* < 0.01). ns indicates no significant difference.

A total of 38 positional candidate genes were detected for *qPEL_GSI_3.2* in the 200 kb chromosome region containing *qPEL_GSI_3.2* (Figure 3A,B). Based on the LD analysis, the LD block region was determined to be 8,668,198–8,779,744 bp, containing 19 candidate genes (Figure 3A,B). Of these, seven contained nonsynonymous SNPs (Supplementary Tables S2 and S3), and the nonsynonymous SNPs of the seven genes were significantly associated with PEL_GSI_ in the GWAS (Supplementary Table S2).

Next, the relative expression levels of the seven candidate genes in the panicle neck at stage 8 of young panicle differentiation were analyzed by qRT-PCR in five accessions with high PEL_GSI_ and five accessions with low PEL_GSI_. The qRT-PCR results showed no significant expression difference among the seven candidate genes except gene *LOC_Os03g15770* (Figure 3D and Figure S5). These results indicate that *LOC_Os03g15770* is a strong candidate gene controlling the PEL_GSI_ trait, although further functional validation, such as CRISPR/Cas9 knockout or overexpression experiments, is needed to confirm its role.

The full length of *LOC_Os03g15770* is 3059 bp, which includes seven exons (six coding exons) and six introns (Figure 3C). The gene *LOC_Os03g15770* encodes a 392 amino acid protein. Significantly associated SNP polymorphisms occurred in different regions, including the upstream, the intron, the exons, the 3′-UTR, and the downstream region, leading to the identification of two haplotypes (Figure 3C). The haplotype Hap 1 was associated with a larger PEL_GSI_, while the haplotype Hap 2 was associated with a smaller PEL_GSI_ (Figure 3E). The SNP site (8,699,906) causes a change from base G to base A at nt 181 in the cDNA sequence, resulting in an amino acid change from valine (V) to isoleucine (I) at amino acid 61. The SNP site (8,700,261) causes a change from base T to base A at nt 356 in the cDNA sequence, resulting in an amino acid change from valine (V) to aspartic acid (D) at amino acid 119. The SNP site (8,700,321) causes a change from base C to base T at nt 416 in the cDNA sequence, resulting in an amino acid change from alanine (A) to valine (V) at amino acid 139. The average PEL_GSI_ value of the 63 accessions carrying the Hap 1 allele were 2.69 ± 0.78%, while those with the Hap 2 allele had an PEL_GSI_ of 1.41 ± 0.46%, representing a highly significant difference between Hap 1 and Hap 2 (Figure 3E). These results indicate that Hap 1 is an elite allele that increases PEL_GSI_. The accessions with the highest PEL_GSI_ values were all carrying Hap 1 while the accessions with the lowest PEL_GSI_ values varied with Hap 2 (Table S4), further supporting the accession between the Hap 1 allele and enhanced GA_3_ sensitivity.

### 2.4. Evolutionary and Protein Structure Analysis of LOC_Os03g15770 in Different Species

Based on the gene annotation results, *LOC_Os03g15770* encodes a receptor-like cytoplasmic kinase (RLCK) family protein. To clarify the evolution of the RLCK family protein across different plants, homologous proteins from 21 representative plant species were further analyzed and used to construct a phylogenetic tree (Supplementary Figure S6A). Table S5 lists the information of 21 representative plants, including locus, chromosome, protein length, and genomic position. The phylogenetic tree showed that LOC_Os03g15770 had 81.24% homology consistency with *Oryza glaberrima*, 74.07% with *Brachypodium distachyon*, and 58.12% with *Zizania latifolia*. Figure S6B shows the phylogenetic tree of the homologous proteins of the tyrosine protein kinase family in *Oryza sativa.* The RLCK family can be divided into fourteen independent types (RLCK, RLCKCK, RLCKDUF, RLCKLRR, RLCKWD40, RLCKEGF/LECTIN, RLCKUBOX, RLCKUSP, RLCKUBQ, RLCKSPERM, RLCKJACALIN, RLCKECH, RLCKLYSM, and RLCKPPR) in *Oryza sativa*, consistent with the report by Vij et al. [30]. The LOC_Os03g15770 protein in this study is an RLCK subfamily protein.

To further clarify the differences in the RLCK gene family from the protein structure, protein modeling analysis was performed for the different species such as *Oryza glaberrima*, *Brachypodium distachyon*, *Melica nutans*, *Alopecurus aequalis*, *Zizania latifolia*, *Zizania palustris*, *Bambusa tulda*, and *Aristida adscensionis* (Supplementary Figure S7). Figure S7A shows the protein structures of nine different species. The main structural domains were included, and the proportions of α-helix, extended strand, β-turn, and random coil are listed in Supplementary Figure S7B. All the species contained the serine/threonine protein kinase domain. *Phragmites australis* has the largest α-helix ratio (33.67%). *Zizania latifolia* has the largest extended strand ratio (21.27%). The random coil ratios of all the species are more than 40%. The RLCK protein family consists of 14 types. Except for the protein sequences of RLCKUSP and RLCKUBOX, which are not suitable for protein modeling, models of the other 12 types are shown in Supplementary Figure S8. LOC_Os11g45540 belongs to the RLCKDUF subfamily protein including three domains, i.e., a Ginkbilobin-2-like domain, a serine/threonine protein kinase and a domain of unknown function. LOC_Os02g42620 and LOC_Os07g31210 both contained only one serine/threonine protein kinase domain, while the remaining proteins contained two different domains (Supplementary Figure S8A). The ratios of α-helix, extended strand, β-turn, and random coil in different subfamily proteins are different (Supplementary Figure S8B). LOC_Os12g01910 belongs to the RLCKPPR subfamily and exhibits the highest α-helix proportion (60.32%) and the lowest extended strand (5.75%). These results mentioned above provide additional evidence for the classification of RLCK subfamily proteins into different types in rice.

## 3. Discussion

In this study, the PEL_GSI_ was evaluated in a panel of 173 rice germplasm accessions. Seven QTLs were identified by GWAS to control PEL_GSI,_ with a major focus on the leading QTL *qPEL_GSI_3.2*, located on chromosome 3. Through LD analysis, gene annotation, non-synonymous mutation screening, and expression profiling, *LOC_Os03g15770* was identified as the key candidate gene (requiring further validation) controlling this trait and which encoded an RLCK family protein. Further functional validation through transgenic approaches and gene editing is warranted to definitively establish the role of *LOC_Os03g15770* in GA_3_ sensitivity. Haplotype analysis revealed that Hap 1 is the elite allele, significantly increasing PEL_GSI_. This study reports a genetic locus and a candidate gene regulating exogenous GA_3_ sensitivity in rice, providing new genetic resources and a theoretical basis for simplified hybrid rice seed production and reduced GA_3_ application costs.

Numerous QTLs and genes controlling rice PEL have been mapped, but the vast majority were detected under natural conditions without exogenous hormone treatment [7,15,21]. Although genes such as *EUI1* and *EUI2* affect internode elongation by modulating GA metabolism, they primarily influence the plant’s response to endogenous GA or GA metabolism levels, rather than sensitivity to exogenously applied GA_3_. In this study, we used PEL_GSI_ as a phenotypic indicator to directly quantify the response of germplasm to exogenous GA_3_. The QTL *qPEL_GSI_3.2* was stable over two years, with as many as 221 significant SNPs, indicating its strong effect and reliability. This QTL does not overlap with any previously reported QTL for PEL or plant height, suggesting it is likely a novel genetic locus specifically regulating exogenous GA_3_ sensitivity.

The currently cloned genes controlling PEL mainly involve GA metabolism (*EUI1*, *EPE1*), GA signal transduction (*DEC1*), or cell wall/phospholipid synthesis (SUI family) [12,24,25,26,29]. In contrast, *LOC_Os03g15770* encodes an RLCK family protein. Its typical serine/threonine protein kinase domain suggests that its function depends on substrate protein phosphorylation. RLCKs are generally involved in plant immunity, cell differentiation, and hormone signaling, but their role in GA response has not been reported. Unlike cloned GA metabolic genes (*EUI1*, *EPE1*) or transcription factors (*DEC1*, *HOX12*), RLCKs do not directly synthesize or degrade hormones but act as central signaling nodes that integrate and amplify upstream signals.

Our qRT-PCR results showed that only the expression level of *LOC_Os03g15770* differed significantly between high- and low-PEL_GSI_ materials, indicating that the expression of this gene is closely related to GA_3_ sensitivity. In addition, amino acid substitutions at positions 61 (V → I), 119 (V → D), and 139 (A → V) in the encoded protein underlie the functional divergence between Hap 1 and Hap 2. Among these, the V119D change, from valine to aspartic acid, may substantially alter kinase activity, substrate binding properties, or protein stability. Given that RLCKs often act as downstream components of receptor complexes, we speculate that LOC_Os03g15770 positively regulates GA_3_ signaling through an unknown phosphorylation cascade, thereby enhancing the elongation response of internode cells to exogenous GA_3_. Based on these findings, we propose the following model: after exogenous GA_3_ is perceived by the GID1 receptor, DELLA proteins are degraded via the SCF complex, thereby releasing downstream transcription factors (GAMYB). This process may simultaneously activate an independent membrane-cytoplasmic signaling branch, in which LOC_Os03g15770 is activated by an unknown upstream receptor kinase, subsequently phosphorylating cytoskeleton regulatory proteins or cell wall loosening factors and ultimately promoting internode cell elongation.

The cloned genes controlling rice PEL can be mainly classified into two categories: GA metabolic genes (*EUI1*, *EPE1*) and transcription regulatory genes (*DEC1*, *HOX12*). *EUI1* (*CYP714D1*) inactivates bioactive GA, and its mutation leads to excessive internode elongation [25,26]; *EPE1* similarly degrades bioactive GA [24]. Mutations in these genes result in endogenous GA accumulation, and their response to exogenous GA_3_ is instead attenuated or saturated. *DEC1* (a zinc-finger transcription factor) suppresses internode elongation [29]; *HOX12* directly activates *EUI1* expression [18]. They indirectly affect GA signaling by regulating transcription of downstream target genes. The SUI family affects cell membrane composition; *PEL9* (flavonol synthase) participates in secondary metabolism [21]. Neither directly participates in GA signaling but influences the physical potential for cell elongation. For genes with unknown function, *ACE1* is specifically expressed in the internode meristem, although its function remains unknown.

The *LOC_Os03g15770* identified in this study encodes an RLCK kinase, belonging neither to GA metabolic enzymes nor to transcription factors, but rather it is a novel signaling component. This suggests a new branch pathway exists. *LOC_Os03g15770* represents a completely different regulatory path—potentially a phosphorylation signaling node downstream of the GA receptor and upstream of cell elongation execution proteins. RLCK family proteins have been extensively studied in plant immunity, but rarely in hormone responses. This finding is to directly associate an RLCK with GA_3_ sensitivity.

In this study, the 63 accessions carrying the Hap 1 allele exhibited an average PEL_GSI_ of 2.69%, significantly higher than the 1.41% observed for the Hap 2. This means that under the same exogenous GA_3_ treatment, Hap 1-type materials achieve a greater increase in PEL—i.e., they are more “sensitive” to GA_3_. In the propagation of sterile lines and hybrid rice seed production, the main purpose of GA_3_ application is to induce fertility restoration and promote panicle neck elongation. If a sterile line itself carries the Hap 1 allele, it could theoretically achieve the same PEL effect with a lower GA_3_ dose, thereby reducing hormone costs, minimizing chemical pollution, and avoiding lodging and pre-harvest sprouting caused by excessive GA_3_ [2].

Although the *eui* gene has been introgressed into widely used sterile lines such as Zhenshan 97A and Peiai 64S, their practical application is limited due to other genetic backgrounds or undesirable linkage drag [6]. In contrast, the Hap 1 allele of *LOC_Os03g15770* is naturally present in the germplasm pool and exhibits a dominant/additive effect, making it more convenient to directly introduce into elite sterile or restorer lines via marker-assisted selection or gene editing, without causing linkage drag. Given that PEL is a quantitative trait, the elite haplotype of *LOC_Os03g15770* can be pyramided with other known PEL genes (*EUI1*, *PEL9*) in the future to develop “low-GA_3_, long-panicle-neck” parents for efficient hybrid seed production.

Phylogenetic analysis showed that LOC_Os03g15770 shares high homology with orthologs from other Poaceae species, including *Oryza glaberrima*, *Brachypodium distachyon*, and *Zizania latifolia* (58.12–81.24%), indicating that this gene is relatively conserved in the grass family, and its function in regulating GA_3_ sensitivity may be transferable to other cereal crops. Protein structure prediction further revealed that RLCK proteins from different species all contain a serine/threonine protein kinase domain, but the proportions of α-helices, β-strands, and random coils differ. In the 14 subtypes of the rice RLCK family, the domain compositions vary considerably among members. For example, the RLCKPPR subtype (LOC_Os12g01910) contains an extremely high α-helix proportion (60.32%), suggesting its possible involvement in more complex protein–protein interactions or membrane-binding functions. These structural differences provide a basis for understanding functional diversification within the RLCK family.

In summary, this study identified the major QTL *qPEL_GSI_3.2* controlling PEL_GSI_ in rice and proposed *LOC_Os03g15770* (an RLCK family protein) as the candidate gene. The elite haplotype Hap 1 significantly enhances GA_3_-induced PEL. This finding not only expands the genetic basis of hormone sensitivity regulation but also provides a new tool for breeding “low-GA_3_-dependent” hybrid rice parents and achieving simplified, environmentally friendly hybrid F_1_ seed production. Further functional validation and breeding applications are warranted.

## 4. Materials and Methods

### 4.1. Plant Materials and Field Planting

The natural population comprised 173 rice accessions previously described by Li et al. [31] (Supplementary Table S6). All 173 accessions were planted in the normal season (13 May) in 2023 and 2024 at the Experimental Station of Anhui Academy of Agricultural Sciences (31°52′ N, 117°17′ E), respectively. Each material planted two replicates with a completely randomized block design. Each accession was planted in two replicates using a completely randomized block design. The space between plants and rows was 20 × 30 cm with routine field management.

### 4.2. GA_3_ Treatment and Phenotypic Investigation

**Figure 4:**
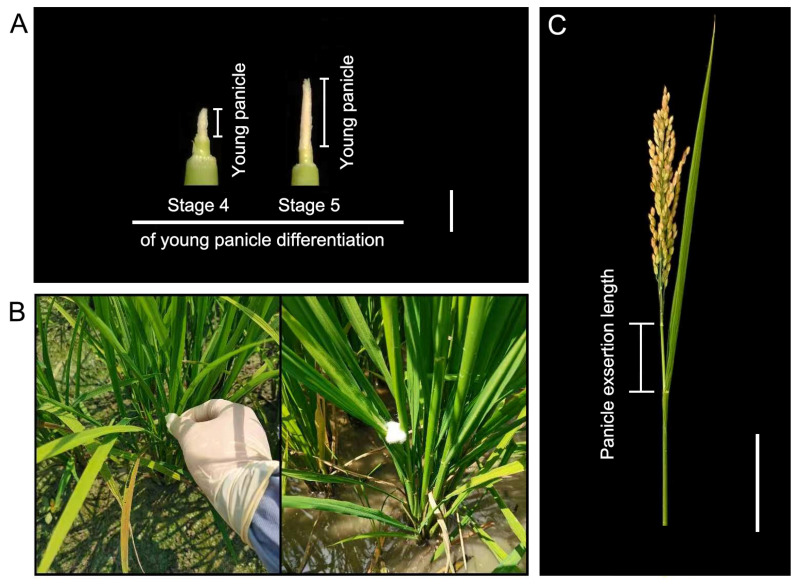
Display of panicle and PEL trait. (**A**) Spikelet morphology at smearing GA_3_. Scale bar, 1 cm. (**B**) Smearing GA_3_ in the field. (**C**) Measurement of PEL trait. Scale bar, 5 cm.

To precisely control the amount of GA_3_, the second leaf from the top at stages 4–5 of young panicle differentiation was smeared with GA_3_ (Figure 4A,B). Stages 4–5 of young panicle differentiation were determined according to the classification described by Itoh et al. [32]. At these stages, the panicle length is approximately 1.0–4.0 cm in length, and the second leaf from the top is fully expanded. This stage corresponds to approximately 50–65 days after transplanting under our local conditions. We chose this developmental window because the uppermost internode is undergoing active cell division and elongation during stages 4–5, when the application of GA_3_ is most effective in promoting internode elongation.

The smearing was performed on the middle portion of the leaf blade using a fine brush. Each accession received 0.1 mg of GA_3_ at a concentration of 0.1 mg/mL. For the control group, an equivalent volume of distilled water was smeared on the second leaf from the top of the same developmental stage using the same method, while all other field management practices remained identical between the two groups. The hormone was applied continuously for 3 days at a ratio of 3:4:3 to ensure even application on the leaves. Twenty-five days after heading, we measured the PEL of the main stem panicle (Figure 4C). Ten plants from each accession were sampled and measured, and the mean PEL was calculated as the phenotypic value. PEL under normal conditions was designated as PELn_,_ and PEL under GA_3_ treatment as PEL_GA3_. PEL_D_ was defined as the difference between PEL_GA3_ and PEL_n._ The GA_3_ sensitivity index (PEL_GSI_) was calculated as PEL_GSI_ = PEL_D_/PEL_n_ and used as the sensitivity indicator for GA_3_ in rice. The average values of PEL_n_, PEL_GA3_, and PEL_GSI_ are listed in Table S6. For accessions with PELn as 0 cm, the PEL_GSI_ value was not defined, and these accessions were excluded from the PEL_GSI_-based GWAS. The number of excluded accessions was fewer than 2 in each environment, accounting for less than 2% of the total population.

### 4.3. Genotypic Data Obtained

We downloaded the sequences of 173 accessions from the NCBI Sequence Read Archive with the accession number PRJNA554986 [33]. The paired-end reads were aligned to the Nipponbare reference genome (IRGSP 1.0) using Bowtie 2 [34]. More than 95% of reads were mapped to the reference genome with a mapping score > 60. SNP calling was performed using the HaplotypeCaller from GATK 3.8-0. Missing genotype data were imputed with Beagle v4.1 [35]. Ultimately, a total of 1,322,884 SNPs were obtained. SNPs with a minor allele frequency (MAF) > 5% and a missing rate < 20% were retained.

### 4.4. Genome-Wide Association Analysis

Based on the software TASSEL 5.2.1 [36], the kinship matrix was calculated. The genome-wide association study (GWAS) was conducted using an efficient mixed-model association expedited [37]. According to Yang et al. [38], a genomic region was considered a QTL when the leading SNP had a *p* value < 1 × 10^−6^ and clear peak-like signals (with more than five significant SNP loci). The QTL region was defined as the 200 kb interval (100 kb upstream and 100 kb downstream) surrounding the leading SNP. The R package “LDheatmap” [39] was used to construct the linkage disequilibrium (LD) heatmap surrounding peaks. The Manhattan and quantile–quantile plots were drawn by qqman [40]. The significance threshold of GWAS was determined as 1.0 × 10^−6^ at a significance level of 0.05 based on the false discovery rate correction method reported by Benjamini and Hochberg [41]. The effective number of independent SNP markers was estimated using the method of Gao et al. [42], yielding approximately 49,000 independent tests and a threshold of 1.0 × 10^−6^.

### 4.5. Quantitative Reverse Transcription PCR Analysis of Candidate Genes

The uppermost internode was sampled at heading stage from five accessions with high PEL_GSI_ (Chenwan 3hao, Chuan 6xian, Haobuka, Guichao 2hao, and Shengyou 2hao) and five accessions with low PEL_GSI_ (Wumangzaodao, Wandao 68, Xudao 25-7, Xiaobaidao, and Qingkong). Total RNA was extracted using the TianGen Pure Plant Plus Kit (TianGen Biotech Co., Ltd., Beijing, China) following the manufacturer’s protocol. The first-strand cDNA was synthesized with random oligonucleotides by the HisScript II Reverse Transcriptase system (Vazyme Biotech Co., Ltd., Nanjing, China). The UBQ gene was used as an internal control. The qRT-PCR was performed by the SYBR Green (Vazyme) using the 96-well thermocycler (Roche Applied Science LightCycler 480, Roche Applied Science, Mannheim, Germany, https://lifescience.roche.com/ accessed on 14 April 2026), following the cycling conditions: (i) denaturation (95 °C, 5 min); (ii) amplification and quantification program (95 °C, 10 s; 60 °C, 30 s;72 °C, 60 s); (iii) melting curve (60–95 °C, 7 min); and (IV) cooling step (40 °C, 2 h). For each sample, we conducted three biological replicate experiments. The primers are listed in Table S7. Transcript levels were calculated using the method of Livak and Schmittgen [43].

### 4.6. Candidate Gene Analysis

Candidate genes within the 200 kb genomic region were predicted using the Rice Genome Annotation Project MSU7 database (http://rice.plantbiology.Msu.edu, accessed on 14 April 2026). LD blocks surrounding significant SNPs were determined with Haploview [44]. Based on gene annotation and expression analysis, the causal gene for each locus was identified.

### 4.7. Haplotype Analysis

Haplotypes of candidate genes were determined using the RiceVarMap (http://ricevarmap.ncpgr.cn/ accessed on 14 April 2026) and China Rice Data Center (https://www.ricedata.cn/ accessed on 13 May 2024) databases. Each haplotype was represented by at least 20 varieties.

### 4.8. Protein Structure and Modeling

The secondary protein structure was predicted with PSIPRED [45]. Three-dimensional protein models were generated using AlphaFold 2 [46] and visualized with PyMOL v3.0 [47].

## Data Availability

The data presented in the study are deposited in the SequenceRead Archive (SRA), NCBI accession number PRJNA554986. The names of the repositories and accession numbers can be found in the Article/Supplementary Material.

## Supplementary Materials

The following supporting information can be downloaded at: https://www.mdpi.com/article/10.3390/plants15132063/s1. Table S1. Analysis of variance for panicle exsertion-related traits in rice across two environments. Table S2. The SNP information in a candidate region of 8.67–8.78 Mb on chromosome 3 for PEL_GSI_. Table S3. Annotation of candidate genes in the 8.67–8.78 Mb interval of chromosome 3. Table S4. Representative rice accessions with extreme PEL_GSI_ values and the distribution of SNP loci *LOC_Os03g15770* in the materials. Table S5. The base information of the RLCK family protein from various species. Table S6. The 173 rice accessions’ information used in this study and the mean phenotypic values of PEL_n_, PEL_GA3_, and PEL_GSI_ in the 2023 and 2024 environments. Table S7. The sequences of primers used for qRT-PCR. Figure S1. QTLs identification for PEL_n_ by GWAS in rice. (A) Manhattan plots for PEL_n_ in 2023. (B) QQ-plot for PEL_n_. (C) Information on the identified QTLs for PEL_n_. (D) Manhattan plots for PEL_n_ in 2024. (E) QQ-plot for PEL_n_. (F) Information on the identified QTLs for PEL_n_. The *Y*-axis indicates negative log10 transformed *p* values, and dots above the red line show the significant SNPs in the QTL region. The black arrows indicate the QTL identified. PEL_n_: panicle exsertion length under normal condition. Figure S2. QTLs identification for PEL_GA3_ by GWAS in rice. (A) Manhattan plots for PEL_GA3_ in 2023. (B) QQ-plot for PEL_GA3_. (C) Information on the identified QTLs for PEL_GA3_. (D) Manhattan plots for PEL_GA3_ in 2024. (E) QQ-plot for PEL_GA3_. (F) Information on the identified QTLs for PEL_GA3_. The *Y*-axis indicates negative log10 transformed *p* values, and dots above the red line show the significant SNPs in the QTL region. The black arrows indicate the QTL identified. PEL_GA3_: panicle exsertion length under GA_3_ condition. Figure S3. QTLs identification for PEL_GSI_ by GWAS in rice. (A) Manhattan plots for PEL_GSI_ in 2023. (B) QQ-plot for PEL_GSI_. (C) Information on the identified QTLs for PEL_GSI_. (D) Manhattan plots for PEL_GSI_ in 2024. (E) QQ-plot for PEL_GSI_. (F) Information on the identified QTLs for PEL_GSI_. The *Y*-axis indicates negative log10 transformed *p* values, and dots above the red line show the significant SNPs in the QTL region. The black arrows indicate the QTL identified. PEL_GSI_: GA_3_ sensitivity index of PEL. Figure S4. QTLs identification for PEL_D_ by GWAS in rice. (A) Manhattan plots for PEL_D_ in 2023. (B) QQ-plot for PEL_D_. (C) Information on the identified QTLs for PEL_D_. (D) Manhattan plots for PEL_D_ in 2024. (E) QQ-plot for PEL_D_. (F) Information on the identified QTLs for PEL_D_. (G) Manhattan plots for PEL_D_ in the whole population of rice accessions. (H) QQ-plot for PEL_D_. (I) Manhattan plots for PEL_D_ in the whole population of rice accessions. The *Y*-axis indicates negative log10 transformed *p* values, and dots above the red line show the significant SNPs in the QTL region. The black arrows indicate the QTL identified. PEL_D_: the difference between PELGA_3_ and PEL_n_. Figure S5. The qRT-PCR results of six candidate genes in different materials. The relative expression values were normalized to the rice UBQ gene. Error bars indicate standard deviation, and n.s. indicates no significant difference. Figure: S6. Phylogenetic analysis of the LOC_Os03g15770 protein. Phylogenetic tree of the complete amino acid sequence from various species (*Lolium perenne*, *Festuca glaucescens*, *Alopecurus aequalis*, *Melica nutans*, *Brachypodium distachyon*, *Oryza glaberrima*, *Oryza sativa*, *Zizania latifolia*, *Zizania palustris*, *Bambusa tulda*, *Aristida adscensionis*, *Phragmites australis*, *Eragrostis australis*, *Hordeum erectifolium*, *Zea mays*, *Urochloa decumbens*, *Panicum virgatum*, *Panicum hallii*, *Sesamum alatum*, and *Arabidopsis thaliana*). (A) Phylogenetic analysis of LOC_Os03g15770 protein in different species. (B) Phylogenetic analysis of LOC_Os03g15770 protein in *Oryza sativa* L. species. Figure S7. The protein structure and basic information statistics of LOC_Os03g15770 homologous protein in different species. (A) The protein structure in different species. The red color indicates the position of the main domain. (B) Basic statistics related to the protein structure in different species. Figure S8. The protein structure and basic information statistics of LOC_Os03g15770 homologous protein in the same species. (A) The protein structure of different types of the same species. The different color indicates the position of different main domains. (B) Basic statistics related to the protein structure of different types of the same species.
