# Supplementary material for: Genome-Wide Association Analysis of Gibberellin Sensitivity for Panicle Exsertion Length in Rice and Candidate Gene Identification
Source: Plants (Basel). 2026 Jul 2;15(13):2063. doi: 10.3390/plants15132063 (PMC13364160; doi:10.3390/plants15132063)
Supplement: Supplementary file 1 [file plants-15-02063-s001.zip › Table S1.pdf]

Table S1. Analysis of variance for panicle exsertion-related traits in rice across two environments.

Traits	Source of variation	df	SS	MS	F value
PEL_n	Block	4	2.54	0.64	1.64
	Environment	1	0.06	0.06	0.16
	Genotype	172	10956.29	63.70	164.69**
	Genotype×Environment	172	132.54	0.77	1.99**
	Error	688	266.11	0.39	
PEL_{GA3}	Block	4	2.01	0.50	1.20
	Environment	1	0.80	0.80	1.91
	Genotype	172	24796.80	144.17	343.73**
	Genotype×Environment	172	10235.32	59.51	141.88**
	Error	688	288.50	0.42	
PEL_{GSI}	Block	4	9.56	2.39	1.77
	Environment	1	0.01	0.01	0.01
	Genotype	172	3425.30	19.91	14.75**
	Genotype×Environment	172	466.01	2.71	2.01**
	Error	688	929.15	1.35	
PEL_D	Block	4	5.21	1.30	1.74
	Environment	1	1.32	1.32	1.76
	Genotype	172	9206.04	53.52	71.55**
	Genotype×Environment	172	234.93	1.37	1.83**
	Error	688	514.66	0.75	

Note: PEL_n, PEL in normal condition; PEL_{GA3}, PEL in GA_3 treatment; PEL_{GSI}, GA_3 sensitivity index of PEL; PEL_D, the difference between PEL_{GA3} and PEL_n; df, degrees of freedom; SS, sum of squares; MS, mean squares.
