# Supplementary material for: Genome-Wide Association Analysis of Gibberellin Sensitivity for Panicle Exsertion Length in Rice and Candidate Gene Identification
Source: Plants (Basel). 2026 Jul 2;15(13):2063. doi: 10.3390/plants15132063 (PMC13364160; doi:10.3390/plants15132063)
Supplement: Supplementary file 1 [file plants-15-02063-s001.zip › Table S3.pdf]

Table S3. Annotation of candidate genes in the 8.67–8.78 Mb interval of chromosome 3.

Genetic symbol	Annotation of gene function	Gene position/bp
LOC_Os03g15720	Expressed protein	8,668,051 - 8,671,138
LOC_Os03g15730	S-RNase binding protein	8,673,598 - 8,676,582
LOC_Os03g15740	Uncharacterized TPR repeat containing protein	8,679,110 - 8,682,336
LOC_Os03g15750	Expressed protein	8,683,553 - 8,687,628
LOC_Os03g15770	Tyrosine protein kinase domain containing protein	8,698,047 - 8,702,758
LOC_Os03g15810	AAA-type ATPase family protein	8,720,361 - 8,728,030
LOC_Os03g15830	Transposon protein	8,732,767 - 8,736,090
