# Supplementary material for: Genome-Wide Association Analysis of Gibberellin Sensitivity for Panicle Exsertion Length in Rice and Candidate Gene Identification
Source: Plants (Basel). 2026 Jul 2;15(13):2063. doi: 10.3390/plants15132063 (PMC13364160; doi:10.3390/plants15132063)
Supplement: Supplementary file 1 [file plants-15-02063-s001.zip › Table S4.pdf]

Table S4. Representative rice accessions with extreme PEL_{GSI} values and the distribution of SNP loci of *LOC_Os03g15770* in the materials.

Material name	SNP loci in *LOC_Os03g15770*															PEL_{GSI}
	8,696,465	8,697,066	8,697,098	8,697,159	8,697,925	8,698,622	8,698,646	8,699,193	8,699,906	8,700,261	8,700,321	8,700,835	8,702,699	8,702,741	8,702,940	
Xudao 4hao	T	A	C	T	T	G	C	A	G	T	C	T	T	T	A	13.50
Yandao 6hao	T	A	C	T	T	G	C	A	G	T	C	T	T	T	A	10.45
Ningjignhui 260	T	A	C	T	T	G	C	A	G	T	C	T	T	T	A	9.55
Huifeng 1	T	A	C	T	T	G	C	A	G	T	C	T	T	T	A	7.37
Nuohangu	T	A	C	T	T	G	C	A	G	T	C	T	T	T	A	7.05
Guihuahuang	T	A	C	T	T	G	C	A	G	T	C	T	T	T	A	5.55
Wuyunjing 21hao	T	A	C	T	T	G	C	A	G	T	C	T	T	T	A	5.55
Ningjinghui 237	T	A	C	T	T	G	C	A	G	T	C	T	T	T	A	5.50
Zhendao 2hao	T	A	C	T	T	G	C	A	G	T	C	T	T	T	A	5.30
Zhongjing 131	G	G	T	C	C	C	T	G	A	A	T	G	C	C	G	0.34
Longjing 8hao	G	G	T	C	C	C	T	G	A	A	T	G	C	C	G	0.33
Yangfujing 4901	G	G	T	C	C	C	T	G	A	A	T	G	C	C	G	0.33
Yuedao 68	G	G	T	C	C	C	T	G	A	A	T	G	C	C	G	0.31
Fengliagnyou 8hao	G	G	T	C	C	C	T	G	A	A	T	G	C	C	G	0.28
Yuedao 48	G	G	T	C	C	C	T	G	A	A	T	G	C	C	G	0.26
Yuedao 66	G	G	T	C	C	C	T	G	A	A	T	G	C	C	G	0.21
Baikenuo	G	G	T	C	C	C	T	G	A	A	T	G	C	C	G	0.13
Yuedao 9	G	G	T	C	C	C	T	G	A	A	T	G	C	C	G	0.10
