# Supplementary material for: Genome-Wide Association Analysis of Gibberellin Sensitivity for Panicle Exsertion Length in Rice and Candidate Gene Identification
Source: Plants (Basel). 2026 Jul 2;15(13):2063. doi: 10.3390/plants15132063 (PMC13364160; doi:10.3390/plants15132063)
Supplement: Supplementary file 1 [file plants-15-02063-s001.zip › Table S5.pdf]

Table S5. The base information of the RLCK family protein from various species.

No.	Locus	Chr.	protein length	Start position	End position	species
1	XP_051182114	4	515	311,861,763	311,866,538	Lolium perenne
2	KAM0891735	1	412	1,487,785	1,490,188	Festuca glaucescens
3	CAM0872432	1	375	111,552,740	111,555,328	Alopecurus aequalis
4	CAN8505305	1	393	88,276,099	88,283,079	Melica nutans
5	KQK22443	1	387	66,143,637	66,147,592	Brachypodium distachyon
6	XP_052147267	3	392	8,236,218	8,239,993	Oryza glaberrima
7	KAL5218711	5	392	28,040,960	28,046,836	Zizania latifolia
8	KAG8096662	13	392	53,370,298	53,372,886	Zizania palustris
9	CAO6222685	3	392	11,906,363	11,916,328	Bambusa tulda
10	KAL6907585	2	393	1,912,277	1,915,271	Aristida adscensionis
11	XP_062216125	4	392	39,543,959	39,548,140	Phragmites australis
12	TVU47647	5	391	36,915,292	36,918,140	Eragrostis curvula
13	CAP3036640	4	382	416,149,432	416,152,254	Hordeum erectifolium
14	NP_001142074	1	393	38,093,323	38,097,101	Zea mays
15	CAL4930264	15b	396	10,865,410	10,867,993	Urochloa decumbens
16	XP_039786808	9k	336	60,023,856	60,027,109	Panicum virgatum
17	RLN28459	5	897	42,343,694	42,352,200	Panicum miliaceum
18	PAN20084	3	947	22,906,652	22,918,208	Panicum hallii
19	KAK4418950	1	378	9,666,640	9,668,814	Sesamum alatum
20	AEE34897	1	809	26,020,089	26,026,285	Arabidopsis thaliana
21	LOC_Os03g15770	3	392	8,698,047	8,702,758	Oryza sativa
22	LOC_Os03g16740	3	374	9,269,491	9,273,582	Oryza sativa
23	LOC_Os02g42620	2	369	25,639,087	25,641,155	Oryza sativa
24	LOC_Os07g31210	7	669	18,479,043	18,481,052	Oryza sativa
25	LOC_Os09g17890	9	326	10,943,486	10,947,944	Oryza sativa
26	LOC_Os01g36500	1	848	20,256,262	20,260,718	Oryza sativa
27	LOC_Os11g45540	11	625	27,571,083	27,574,113	Oryza sativa
28	LOC_Os03g05140	3	1030	2,495,806	2,498,898	Oryza sativa
29	LOC_Os04g58910	4	938	35,039,915	35,043,790	Oryza sativa
30	LOC_Os11g10640	11	715	5,835,229	5,841,560	Oryza sativa
31	LOC_Os11g10710	11	402	5,873,795	5,880,713	Oryza sativa
32	LOC_Os04g24510	4	683	14,056,400	14,061,288	Oryza sativa

33	LOC_Os03g44050	3	753	24,759,259	24,763,495	Oryza sativa
34	LOC_Os03g31000	3	692	17,660,012	17,667,505	Oryza sativa
35	LOC_Os06g04880	6	806	2,140,038	2,146,619	Oryza sativa
36	LOC_Os01g39970	1	765	22,538,988	22,542,228	Oryza sativa
37	LOC_Os02g05820	2	775	2,869,670	2,874,218	Oryza sativa
38	LOC_Os11g18670	11	353	10,548,638	10,556,200	Oryza sativa
39	LOC_Os10g12620	10	795	7,022,929	7,031,173	Oryza sativa
40	LOC_Os11g17380	11	693	9,688,431	9,695,220	Oryza sativa
41	LOC_Os04g30030	4	463	17,913,437	17,916,778	Oryza sativa
42	LOC_Os11g39490	11	837	23,508,365	23,513,127	Oryza sativa
43	LOC_Os11g17440	11	1035	9,719,961	9,728,087	Oryza sativa
44	LOC_Os11g34624	11	596	20,264,311	20,275,890	Oryza sativa
45	LOC_Os12g01910	12	887	531,180	535,127	Oryza sativa
