# Supplementary material for: Genome-Wide Association Analysis of Gibberellin Sensitivity for Panicle Exsertion Length in Rice and Candidate Gene Identification
Source: Plants (Basel). 2026 Jul 2;15(13):2063. doi: 10.3390/plants15132063 (PMC13364160; doi:10.3390/plants15132063)
Supplement: Supplementary file 1 [file plants-15-02063-s001.zip › Table S7.pdf]

Table S7. The sequences of primers used for qRT-PCR.

Gene name	Forward primer sequence (5'-3')	Reverse primer sequence (5'-3')
UBQ	ACCCTGGCTGACTACAACATC	AGTTGACAGCCCTAGGGTG
LOC_Os03g15720	ACCTTCTGTTCCATGTGCTTC	GGCCCACCATCCCATTTCTT
LOC_Os03g15730	AGGCAAAGCAATTTGAGGCG	TAGTTGCAAATTGGCGGCAC
LOC_Os03g15740	ATCACATGGTCATCGGCAGG	AGTATGGCAGCCTCTCGGTA
LOC_Os03g15750	AGCTGCACATTGCAAGAAAAGA	GACCGCCAAACTGTGCAAAT
LOC_Os03g15770	GCTGAATCAAAGCAAGGGGC	CCTTGGTCCTGATCACAAAGGT
LOC_Os03g15810	TGTCACATTCTGCAGGGAGG	TGCACTAATCCACTGCAGGC
LOC_Os03g15830	ATTGGCTGCAGCTATGCTCT	GTGCAGTCCTCCTTCGAGAC
