# Supplementary figures and images for: Genome-Wide Association Analysis of Gibberellin Sensitivity for Panicle Exsertion Length in Rice and Candidate Gene Identification
Source: Plants (Basel). 2026 Jul 2;15(13):2063. doi: 10.3390/plants15132063 (PMC13364160; doi:10.3390/plants15132063)

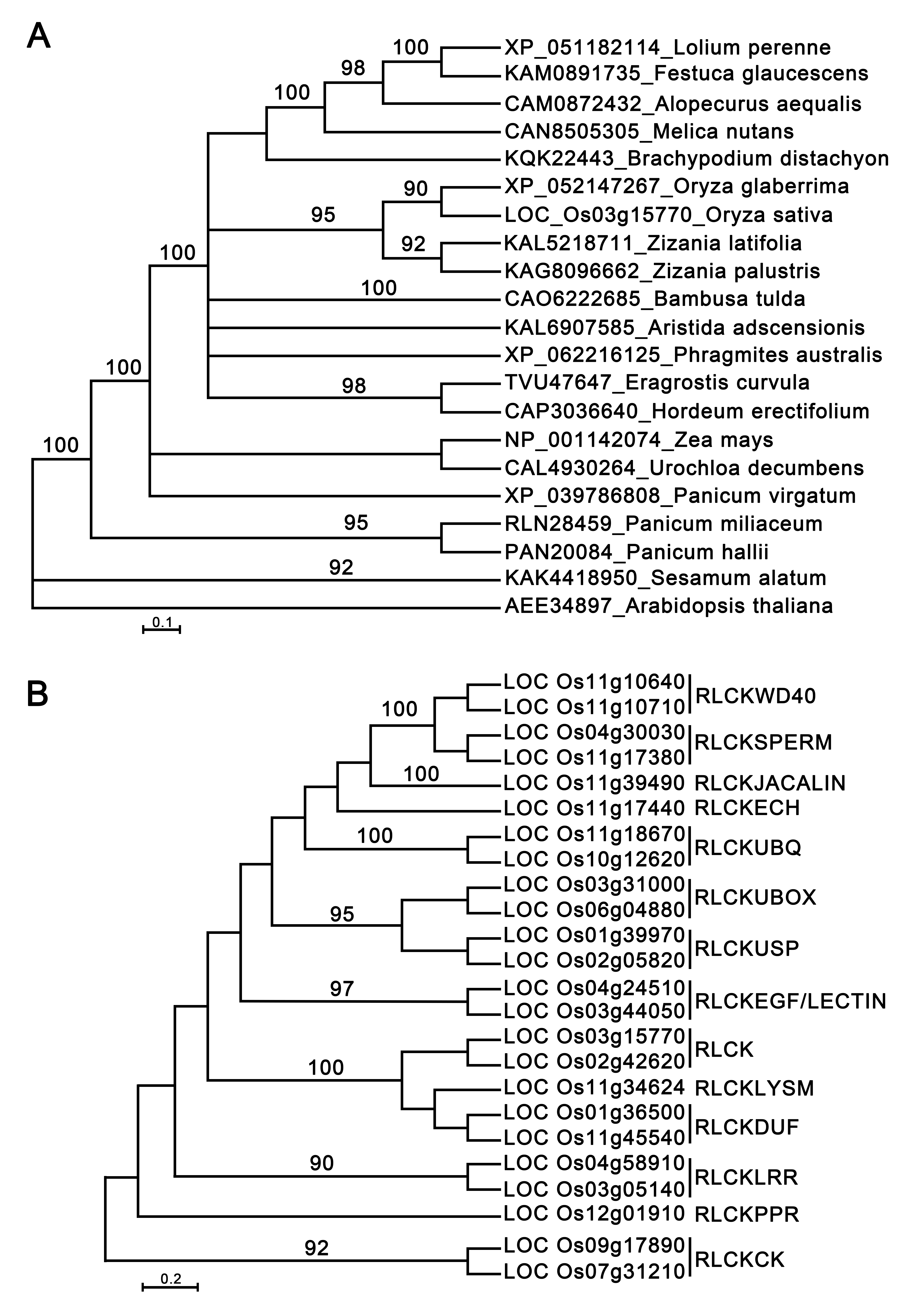

Supplement: Supplementary file 1 [file plants-15-02063-s001.zip › Figure S6.tif]

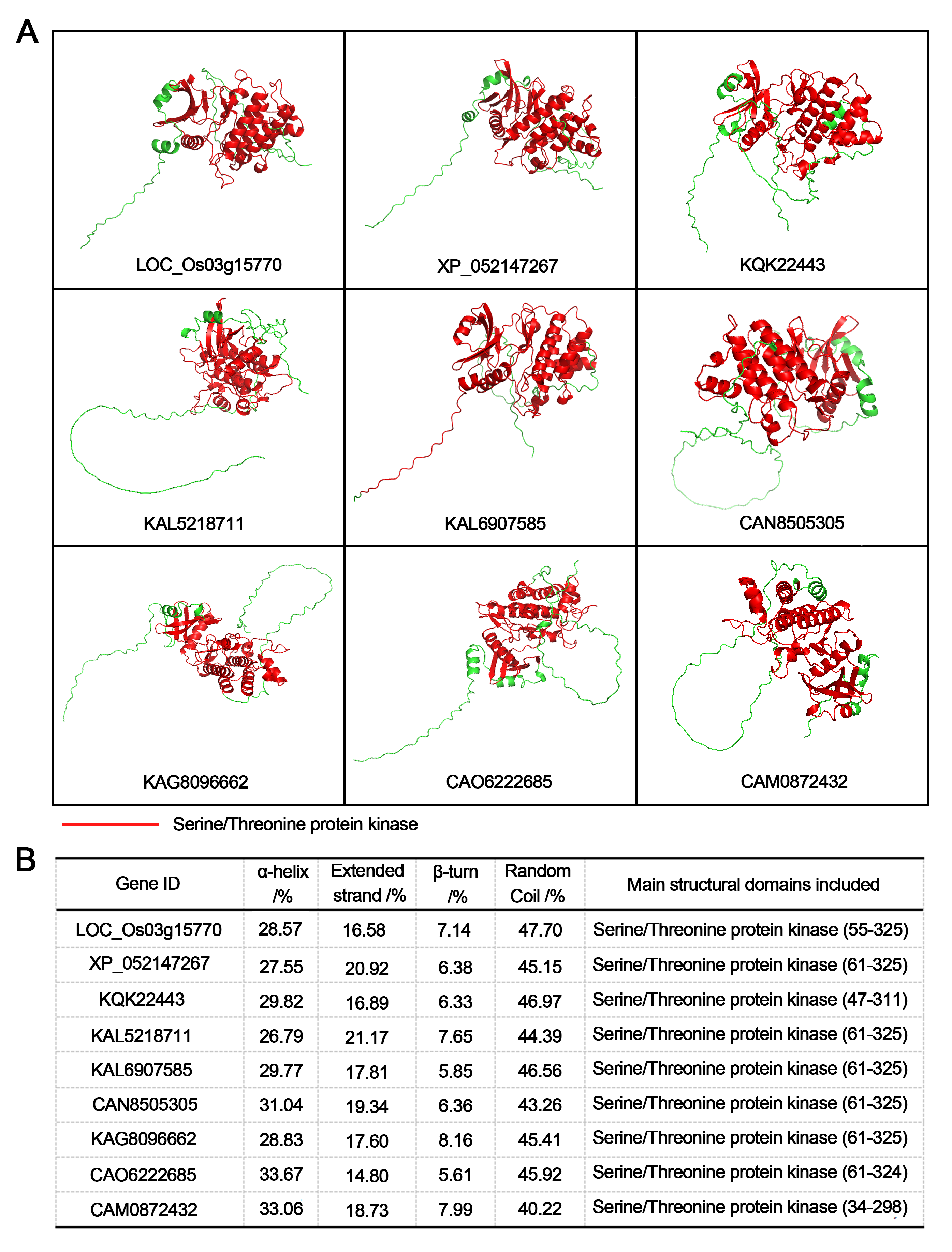

Supplement: Supplementary file 1 [file plants-15-02063-s001.zip › Figure S7.tif]

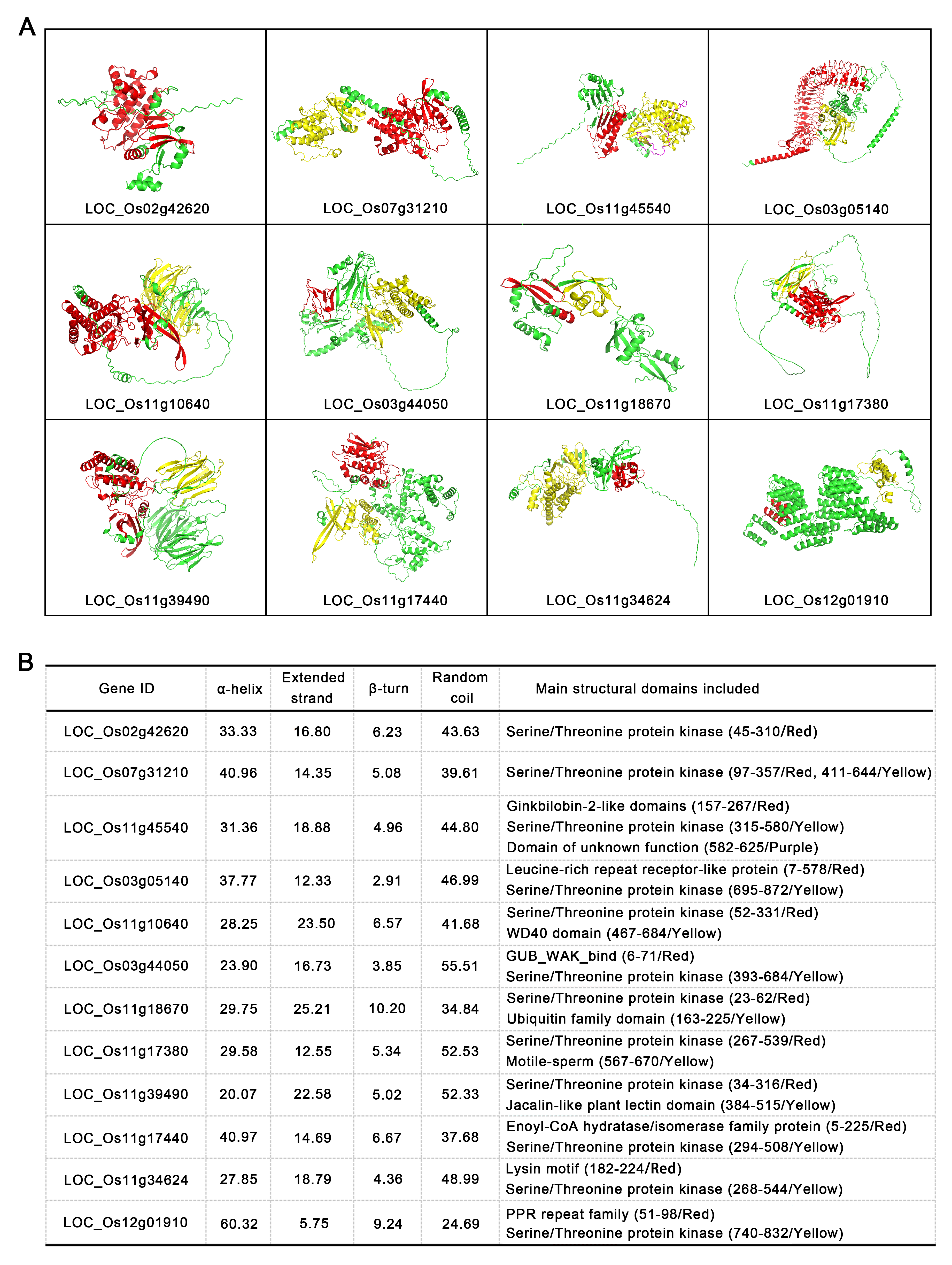

Supplement: Supplementary file 1 [file plants-15-02063-s001.zip › Figure S8.tif]
